# Vacuolar Proteases of *Candida auris* from Clades III and IV and Their Relationship with Autophagy

**DOI:** 10.3390/jof11050388

**Published:** 2025-05-18

**Authors:** Daniel Clark-Flores, Alvaro Vidal-Montiel, Ricardo Mondragón-Flores, Eulogio Valentín-Gómez, César Hernández-Rodríguez, Margarita Juárez-Montiel, Lourdes Villa-Tanaca

**Affiliations:** 1 Laboratorio de Biología Molecular de Bacterias y Levaduras, Departamento de Microbiología, Escuela Nacional de Ciencias Biológicas, Instituto Politécnico Nacional, Prol. de Carpio y Plan de Ayala. Col. Sto. Tomás, Ciudad de México 11340, Mexico; clark3dd@gmail.com (D.C.-F.); alvaro301013@gmail.com (A.V.-M.);; 2 Departamento de Bioquímica, Centro de Investigación y de Estudios Avanzados del Instituto Politécnico Nacional (CINVESTAV-IPN), Av. IPN No. 2508, Ciudad de México 07360, Mexico; rmflores@cinvestav.mx; 3 Departmento de Microbiología y Ecología, Universidad de Valencia, 46100 Valencia, Spain; eulogio.valentin@uv.es; 4 Severe Infection Research Group, Health Research Institute La Fe, 46026 Valencia, Spain

**Keywords:** *Candida auris*, *Candidozyma auris*, autophagy, vacuolar proteases, peptidases, nutritional stress, rapamycin, Atg8

## Abstract

*Candida auris* is a multidrug-resistant pathogen with a high mortality rate and widespread distribution. Additionally, it can persist on inert surfaces for extended periods, facilitating its transmissibility in hospital settings. Autophagy is a crucial cellular mechanism that enables fungal survival under adverse conditions. A fundamental part of this process is mediated by vacuolar proteases, which play an essential role in the degradation and recycling of cellular components. The present work explores the relationship between *C. auris* vacuolar peptidases and autophagy, aiming to establish a precedent for understanding the survival mechanisms of this emerging fungus. Thus, eight genes encoding putative vacuolar peptidases in the *C. auris* genomes were identified: *PEP4*, *PRB1*, *PRC1*, *ATG42*, *CPS*, *LAP4*, *APE3*, and *DAP2*. Analysis of the protein domains and their phylogenetic relationships suggests that these enzymes are orthologs of *Saccharomyces cerevisiae* vacuolar peptidases. Notably, both vacuolar protease gene expression and the proteolytic activity of cell-free extracts increased under nutritional stress and rapamycin. An increase in the expression of the *ATG8* gene and the presence of autophagic bodies were also observed. These results suggest that proteases could play a role in yeast autophagy and survival during starvation conditions.

## 1. Introduction

*Candida auris* (syn. *Candidozyma auris*) is a multidrug-resistant pathogenic yeast with a widespread distribution, high mortality (41%), and high transmissibility [1]. *C. auris* isolates are classified into six clades based on genome structure and geographical distribution. Phenotypic and genotypic differences among these clades have been reported, including antifungal resistance, survival capacity, virulence, metabolism, cell wall composition, and immune system interactions [2,3,4,5,6,7,8]. Additionally, several clade-specific putative virulence factors, such as adhesins, lipases, and proteases, have been proposed [9].

Proteases are enzymes capable of cleaving the peptide bond and are classified based on different characteristics, such as: (1) the pH at which they act as acidic, neutral, or alkaline; (2) the catalytic residues in their active site as aspartyl, serine, cysteine peptidases; and (3) the type of reaction they perform as endopeptidases or exopeptidases [10]. The endopeptidases can cleave within the internal structure of the protein, while exopeptidases can remove amino acids from one of the ends of the protein. Exopeptidases can be further classified as aminopeptidases or carboxypeptidases if they act at the amino or carboxyl end peptide bond, respectively. The aminopeptidases can also be classified as dipeptidyl aminopeptidases, which catalyze the sequential release of dipeptides from the amino end [11].

In *Candida* spp., aspartyl peptidases, such as secreted aspartyl proteases (SAPs), cell wall-anchored aspartyl peptidases (yapsins), and vacuolar proteases, play various cellular functions. Vacuolar proteases are nonspecific enzymes residing in the vacuolar lumen or membrane, primarily responsible for degrading senescent or non-functional proteins and organelles through autophagy [12,13,14]. *Saccharomyces cerevisiae* has been an excellent model for the study of vacuolar proteases and their role in autophagy, a conserved eukaryotic survival mechanism that ensures cellular viability under stress conditions, such as nutrient starvation [15,16,17,18]. In *S. cerevisiae*, at least eight vacuolar peptidases have been characterized well: an acidic aspartyl endopeptidase (PrA), a neutral serine endopeptidase (PrB), two serine carboxypeptidases (CpY and Atg42), a metallo-carboxypeptidase (CpS), two metallo-aminopeptidases (Ape1 and Ape3), and one serine dipeptidyl aminopeptidase (Dap2) [15].

Autophagy is regulated by the Ser/Thr kinase target of rapamycin (TOR), which inhibits various autophagy-related proteins (Atg) through phosphorylation. Under starvation conditions, TOR is inactivated, leading to the dephosphorylation of key Atg proteins and the initiation of autophagy. This process begins with *de novo* formation of a double-membrane structure called a phagophore, which sequesters cellular components either selectively or non-selectively. The phagophore subsequently matures into an autophagosome upon Atg8 phosphorylation. The autophagosome then migrates and fuses with the vacuole, forming an autophagic body, which is finally degraded by vacuolar hydrolases to generate energy or cellular component replacement, thereby promoting cell survival [18].

Vacuolar peptidases have been studied in different models of human and plant pathogenic fungi, where their importance in the survival, virulence, and dimorphism process has been suggested [14,19]. However, their function in *C. auris* remains unknown. Investigating these enzymes could enhance our understanding of autophagy in pathogenic yeasts. In this study, we demonstrate that *C. auris* exhibits increased enzymatic activity of vacuolar proteases, upregulation of vacuolar protease-encoding genes, and accumulation of autophagic bodies under nutritional stress and rapamycin-induced autophagy.

## 2. Materials and Methods

### 2.1. Strains, Media, and Growth Conditions

For this study, *C. auris* CJ97, from Hospital La Fe, Valencia, Spain, and *C. auris* 20-1498, donated by Dr. Gloria Gonzalez from Universidad Autónoma de Nuevo Léon, México, were used as model organisms. To assess the enzymatic activity and gene expression of putative vacuolar peptidases and *ATG8*, both strains were grown separately in different culture media under controlled conditions. The culture media include YPD broth (1% yeast extract, 2% peptone, and 2% dextrose; Sigma-Aldrich, St. Louis, MO, USA; Y1375), Yeast Nitrogen Base medium (YNB; USBiological, Swampscott, MA, USA; C7053116) supplemented with 2% dextrose (Sigma-Aldrich, St. Louis, MO, USA; D9434) and 0.5% ammonium sulfate (Gibco, Waltham, MA, USA; 895-1051IP) (YNB+C+N), YNB with 2% dextrose (YNB+C−N), YNB with 0.5% ammonium sulfate (YNB−C+N), YNB without supplements (YNB−C−N), and YNB+C+N supplemented with 5 nM of rapamycin (Sigma-Aldrich, St. Louis, MO, USA; 37094) (YNB+C+N+Rap).

Enzymatic activity assays and gene expression analysis were assessed for non-proliferating cultures. Therefore, the growth phases of each *C. auris* strain were determined by inoculating the yeasts separately in YPD medium at an initial OD_600_ of 0.05 and incubating at 37 °C under constant shaking (100 rpm). The OD_600_ was measured every two hours for 48 h, revealing that both strains reached the early stationary phase after 15 h of incubation. At this growth phase, 25 mL of *C. auris* cultures was harvested from YPD medium. Yeast cells were washed twice with fresh YNB+C+N medium and then resuspended in 25 mL of YNB broth with the respective treatment. Cultures were incubated at 37 °C under constant shaking (100 rpm) for six hours.

### 2.2. Determination of Specific Protease Activity

Cells were harvested from the different culture media via centrifugation at 10,000 rpm for 5 min at 4 °C. The resulting pellets were homogenized and lysed using a FAST-Prep-24 system (MB Biomedicals, Santa Ana, CA, USA) with an equal volume of sterile glass beads (0.425–0.6 mm diameter; Sigma-Aldrich, St. Louis, MO, USA; G9268). Lysis was performed in three pulses at 6.5 m/s for 30 s each, with intervals of one minute on ice. Afterward, a volume of cold 100 mM Tris-HCl buffer (pH 7.6; Promega, Madison, WI, USA; H1523) was added, followed by a final FAST-Prep-24 pulse to ensure a complete cell disruption. Cell lysis was confirmed by microscopy, and cell-free extract was obtained by centrifugation at 10,000 rpm for 10 min at 4 °C. Cell-free extracts were aliquoted and stored at −70 °C until usage.

Enzymatic activity was assessed using the obtained cell-free extracts, following a previously described methodology [20,21]. Specific activity was expressed as enzyme units per milligram of protein per unit time.

The substrate used to determine the enzymatic activity of acid endoprotease was acid-denaturing hemoglobin (MP Biomedicals, Irivine, CA, USA; ICN90008020). For neutral endoprotease, Hide Powder Azure (HPA; Sigma-Aldrich, St. Louis, MO, USA; H6268) was used. For carboxypeptidase, N-benzoyl-tyrosine-p-nitroanilide (Sigma-Aldrich, St. Louis, MO, USA; B6760) was used. For aminopeptidase, Lys-p-nitroanilide (Bachem, Torrance, CA, USA; LD376) was used, and for dipeptidylpeptidase, Ala-Pro-p-nitroanilide was used (Bachem, Torrance, CA, USA; L1215).

### 2.3. Effect of Peptidase Inhibitors

Cell-free extract was pre-incubated for 30 min at 37 °C in the presence of each inhibitor before the determination of specific protease activity [22]. Residual protease activity was expressed as a percentage, with respect to 100% of activity corresponding to the control reaction without inhibitors. The inhibitors tested included pepstatin A at concentrations of 2.5 μM, 5 μM, and 25 μM (ChemCruz, Dallas, TX, USA; 45036); PMSF at concentrations of 1 mM and 5 mM (Sigma-Aldrich, St. Louis, MO, USA; P7626); Bestatin at concentrations of 100 and 250 μM (Sigma-Aldrich, St. Louis, MO, USA, 58970766); EDTA at concentrations of 1 and 10 mM (J.T. Baker; Radnor, PA, USA; 8993); 1,10-phenanthroline at concentrations of 2.5 mM and 7.5 mM (Sigma-Aldrich, St. Louis, MO, USA; P9375); and E-64 at concentrations of 1 and 10 μM (Sigma-Aldrich, St. Louis, MO, USA; 66701).

### 2.4. RNA Extraction and cDNA Synthesis

Total RNA was extracted from yeast cultures using the hot phenol method [23]. The samples were stored at −70 °C in DEPC-treated water until further use. Genomic DNA contamination was eliminated using the DNase I, RNase free kit (ThermoFisher, Waltham, MA, USA; EN0521). RNA concentration and quality (A_260_/A_280_ ratio) were determined by spectrophotometry, and RNA integrity was verified by electrophoresis on a 1.8% agarose gel (Cleaver Scientific, Rugby, Warwickshire, UK; 18197). cDNA synthesis was performed using RevertAid Reverse Transcriptase (ThermoFisher, Waltham, MA, USA; EP0441) using an oligo(dT)18 primer (ThermoFisher, Waltham, MA, USA; SO131), following the manufacturer’s instructions. cDNA samples were stored at −70 °C until use.

### 2.5. RT-qPCR

Gene expression analysis was performed using RT-qPCR with specific design primers for each putative protease: *PEP4* (Fw: GCTATGACGAGTCCCACTTC, Rv: ATCAACAGAGTACCGGTGTC), *PRB1*(Fw: AGTACGTTGCTGAGTTGTT, Rv: TGCGACTCAAACTTCTTATGAC), *PRC1* (Fw: CCATACTACAAGAACGTGATTG, Rv: TGCGACTCAAACTTCTTATGAC), *LAP4* (Fw: ATATGGCCACAGATTCAAAG, Rv: TAGTACGAGAACCAGTCTGTACG), *DAP2* (Fw: CAGTCTCAATCTTCTTGACGAC, Rv: ATTCTATCAAATACAACAACATTGC), and *ATG8* (Fw: AGGARATCGACAAGMGMAAG, Rv: GGGTGGCAAGATGTCATTG).

RT-qPCR reactions were performed in triplicate in two biological experiments using SYBR-GREEN select Master-Mix (ThermoFisher, Waltham, MA, USA; 4472908) in a thermal cycler (Corbett Research RG-6000; Corbett Robotic Inc., San Francisco, CA, USA). The conditions used were initiation at 95 °C for 5 min, denaturation at 95 °C for 30 s, annealing at 59 °C for 30 s, and extension at 72 °C for 1 min 15 s. Forty-five cycles of denaturation, annealing, and extension were performed. The temperature for the melt curve was from 55 °C to 99 °C. The data were analyzed with 2^ΔΔct^ methodology [24] using *ACT1* as the endogenous gene (Fw: GAAGGAGATCACTGCTTTAGCC, Rv: GAGCCACCAATCCACACAG) [25].

### 2.6. Microscopy of C. auris by TEM

For sample preparation, early stationary-phase *C. auris* cells were treated as mentioned in the growth conditions section. However, cultures were incubated for 11 h. Additionally, samples treated with 5 nM of rapamycin (YNB+C+N+Rap) were supplemented with pepstatin A (2.5 µM) or PMSF (1 mM) and incubated for three more hours, reaching a total incubation time of 14 h.

Cells were harvested and washed with PBS (2000 rpm for 5 min, and resuspended in 3% glutaraldehyde (EMS, Hatfield, PA, USA) for two h at room temperature (RT). Subsequently, cells were washed with PBS and fixed with 1% osmium tetraoxide (EMS, Hatfield, PA, USA) for one hour at RT, followed by one hour at 4 °C. Samples were washed with distilled H_2_O. Block staining with 0.1% filtered aqueous uranyl acetate was performed. Ethanol dehydration was performed at 50%, 60%, and 70% for 10 min at RT, followed by 80%, 90%, and 100% three times for 15 min each. The samples were infiltrated with Spurr’s resin and polymerized at 60 °C for 48 h. Thin sections were obtained using an ultramicrotome (Reichert-Jung, Mount, Waverly, Australia), placed on 200-hole copper grids with a polyvinyl film (polyvinyl formal power; Polysciences Inc. Warrington, PA, USA), and counterstained with 2.5% uranyl acetate in ethanol–water for 30 min at RT and then with saturated lead citrate (aqueous) for 5 min. The samples were visualized under a transmission electron microscope (JEM-1400X at 80 keV, JEOL Ltd., Tokyo, Japan).

Parameters such as cell dimensions, cell wall thickness, and vacuole size were measured using Fiji software v1.54p [26].

### 2.7. Bioinformatic Analysis

Amino acid sequences were retrieved from *Saccharomyces* genome database (https://www.yeastgenome.org) using the following protein IDs: PrA (YPL154C), PrB (YEL060C), CpY (YMR297W), CpS (YJL172W), Atg42 (YBR139W), Ape1 (YKL103C), Ape3 (YBR286W), Dap2 (YHR028C), and Atg8 (YBL078C). Their orthologs were identified using BLASTp searches in five *C. auris* genomes deposited in the NCBI, one representative per clade: clade I (strain 6684; GCA_001189475.1), clade II (strain B11220; GCF_003013715.1), clade III (strain B11221; GCA_031357565.2), clade IV (strain B11243; GCA_003014415.1), and clade V (strain IFRC2087; GCA_016809505.1).

Additionally, ortholog searches were performed using Hidden Markov Models (HMM) in two *C. auris* strains sequenced and annotated by our group: CJ97 (clade III) and 20-1498 (clade IV; GCA_034640365.1) [27].

The best amino acid substitution model was selected using ProtTest v2.4.0, and a phylogenetic tree was constructed with IQTree v2.4.0 [28,29]. Sequence similarity analysis was performed using LOGO 3.0 and Matgat v2.01 [30,31]. Signal peptide prediction was conducted using SignalP 6.0, while domain searches were carried out using Prosite (12 June 2024; https://prosite.expasy.org) and TMHMM v2.0 [32,33]. Additional protein characteristics, including molecular weight, isoelectric point, and amino acid composition, were determined using different Expasy tools (10 June 2024; https://www.expasy.org). Tertiary structure modeling was performed with AlphaFold v2.0 using different templates. Structural visualization and overlay were conducted in USFC Chimera [34]. The quality of the predicted structures was assessed using Ramachandran plot analysis, ERRAT, and VERIFY3D through the SAVESv6.1 server (https://saves.mbi.ucla.edu). Additionally, the prediction of transcription factor binding sites within the 1000 bp upstream promoter regions of each gene encoding putative vacuolar proteases in *C. auris* was performed using the YEASTRACT server (31 January 2025; https://yeastract.com/index.php) [35].

### 2.8. Data Analysis

Statistical analysis was performed using Prism v9, employing two-way ANOVA followed by Tukey’s post hoc test. A confidence value of *p* < 0.05 was considered statistically significant.

## 3. Results

### 3.1. C. auris Orthologs of S. cerevisiae Vacuolar Peptidases

Two clinical isolates of *C. auris* from different clades were used in this work: *C. auris* CJ97 (clade III), originally isolated in Spain [36], and *C. auris* 20-1498 (clade IV), the first Mexican clinical isolate [37]. The inclusion of two *C. auris* strains from different clades was based on increasing reports of clade-specific phenotype variations and genetic polymorphism in *C. auris*.

Amino acid sequences of the proteases PrA, PrB, CpY, CpS, Atg42, Ape1, Ape3, and Dap2 from *S. cerevisiae* were retrieved, and their putative orthologs were identified in the *C. auris* genomes using Hidden Markov Models. A phylogenetic tree was constructed for each putative protease using the sequences retrieved from *C. auris*, including strains from clades I to V, as well as related species from the family *Metschnikowiaceae* and the WGD clade (Figures S1–S4). All *C. auris* sequences were grouped in the same clade, with the putative proteases of the *Candida haemulonii* group as their closest neighbors.

The characteristics of the identified genes and their predicted vacuolar proteases, including length, molecular mass, identity, and sequence similarity, were determined for the clade III and clade IV strains (Table 1). Gene and protein lengths were identical between the two strains, except for Dap2. The percentage of identity and similarity between the aminoacidic sequences from CJ97 and 20-1498 was as follows: 99% and 100% for PrA, 99.6% and 100% for PrB, 98.2% and 99.6% for CpY, 98.3% and 99.8% for CpS, 96.2% and 99.8% for Atg42, 98.7% and 100% for Ape1, 98.5% and 100% for Ape3, and 98.2% and 93.7% for Dap2.

A comparison was also made between the sequences of the study strains and those deposited in the NCBI from the other clades. The comparative analysis revealed similarity percentages above 80% for all cases, except for the CpS protease, which showed a similarity percentage above 60%. Furthermore, sequence alignments revealed no significant amino acid changes within the catalytic domain, while amino acid substitutions in other regions were primarily conservative (Figures S1–S4). Based on these findings, subsequent bioinformatics analysis focused on the *C. auris* clade IV 20-1498.

Further comparative analysis was performed between the *C. auris* 20-1498 proteases and their *S. cerevisiae* orthologs, revealing the following identity and similarity percentages: PrA, 65.5% and 78.2%; PrB, 47.9%, and 61.1%; CpY, 62.2%, and 74.2%; Atg42, 48.6%, and 65.6%; CpS, 38.9%, and 60.2%; Ape1, 52.1%, and 67.1%; Ape3, 50.5%, and 66.5%; and Dap2, 42.0%, and 61.8%.

**Figure 1:**
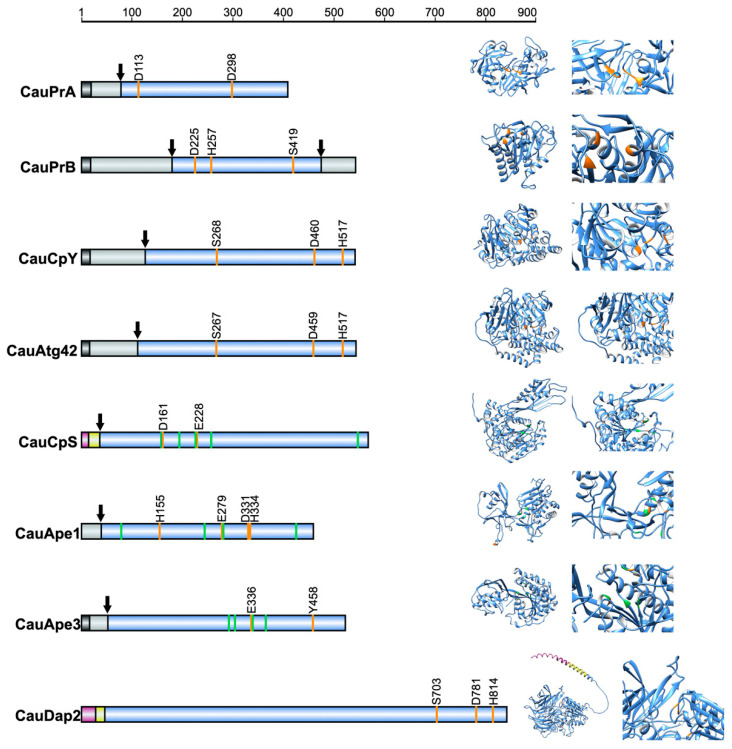
Domains and tertiary structure of putative vacuolar peptidases from *C. auris.* The left panel illustrates the primary structure and domain organization of *C. auris* vacuolar proteases. The signal peptide is depicted in black, the pro-peptide in gray, the cytoplasmic region domain in pink, the transmembrane domain in yellow, the mature protein in blue, the catalytic residues in orange, and the predicted metal cofactor-binding residues in green. The arrows indicate proteolytic cleavage sites. The right panel displays the tertiary structure models for each protease, including close-up views of their catalytic sites.

In *S. cerevisiae*, some vacuolar peptidases are synthesized as pre-pro-peptidases, where the -pre prefix indicates the presence of a signal peptide and the -pro prefix denotes a pro-peptide, which is cleaved by proteases PrA and PrB upon reaching the vacuolar lumen [15]. In *C. auris*, we predicted that PrA, PrB, CpY, Atg42, and Ape3 present both a signal peptide and a pro-peptide (Figure 1). Ape1 was predicted to have only a pro-peptide, while CpS and Dap2 exhibited cytoplasmic and transmembrane domains.

The putative catalytic residues for each predicted protease were also identified. PrA contained two aspartic acid residues in its catalytic motifs, D^113^ and D^298^. PrB exhibited a catalytic triad consisting of D^225^, H^257^, and S^419^. CpY and Atg42 have three catalytic residues, S^268^, D^460^, H^517^ and S^267^, D^459^, and H^517^, respectively. CpS has two catalytic residues, D^161^ and E^228^, and five potential zinc binding residues, H^159^, D^194^, E^229^, D^257^, and H^547^. Ape1 featured four putative catalytic residues, H^155^, E^279^, D^331^, and H^334^, and five zinc-binding residues, H^79^, D^244^, E^280^, D^331^, and H^425^, with E^280^ and D^331^ located in the catalytic domain. Ape3 exhibited five metal-binding residues, H^292^, D^304^, E^337^, D^365^, and H^459^, and two residues potentially involved in the enzymatic reaction, E^336^ and Y^458^, as electron acceptors. Finally, Dap2, predicted to be a serine peptidase, had a catalytic triad composed of S^703^, D^781^, and H^814^.

Three-dimensional structural models of the putative *C. auris* enzymes are shown in Figure 1, with magnified views of their catalytic sites. The tertiary structure of crystallized *S. cerevisiae* vacuolar proteases, PrA (PDB: 1DP5), CpY (PDB: 1CPY), and Ape1 (PDB: 4R8F), were overlaid with their corresponding *C. auris* orthologs (Figure S5). The calculated root mean square deviation (RMSD) values were 0.574 for PrA, 0.647 for CpY, and 0.595 for Ape1.

**Figure 2:**
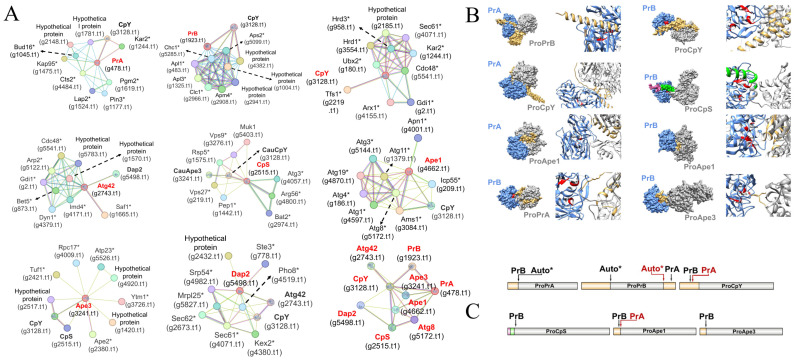
Interactome of vacuolar proteases of *C. auris* and their predicted maturation sites. (**A**) Protein–protein interaction networks of *C. auris* vacuolar proteases. Edges represent different types of associations. Known interactions are represented in light blue, while those recovered from curated databases and experimental determinations are shown in pink. Neighborhood gene interactions are shown in green, gene fusions in red, and gene co-occurrence in dark blue. Light green, black, and cyan represent text mining, co-expression, and homology data in that order. Asterisks (*) denote hypothetical proteins that may be orthologous to *S. cerevisiae* proteins. The proteins studied in this work are highlighted in red. The confidence cutoff point for showing interaction links was set at a high confidence level (0.700). (**B**) Molecular docking of mature protease A or B (blue) interacting with the pro-peptides (yellow) of vacuolar pro-proteases (gray). Theoretical catalytic residues are indicated in red. The predicted cytoplasmic and transmembrane domains of the putative CpS protease from *C. auris* are shown in pink and green, respectively (**C**) Predicted model of vacuolar proteases maturation, including auto-processing mechanism (*). Black and red arrows indicate the proteolytic cleavage sites for PrA and PrB, respectively. The pro-peptide is represented in yellow, the pro-protease in gray, the cytoplasmic domains in pink, and the transmembrane domains in green.

To predict potential protein interaction networks of *C. auris* vacuolar proteases, STRING database analysis was performed, identifying ten proteins associated with *C. auris* peptidases (Figure 2A). Interaction networks were generated for each enzyme separately, as well as for all proteases combined and for the Atg8 protein. In all cases, at least one peptidase interacted with another peptidase, except CpY. Additionally, predicted interactions with transport-related proteins and autophagy-related proteins were observed.

Additionally, the molecular docking analysis revealed that the catalytic residues of *C. auris* PrA and PrB interact with residues located within their own pro-peptides or the pro-peptides of other vacuolar pro-peptidases (Figure 2B,C).

### 3.2. Proteolysis Under Nutritional Stress

Specific intracellular proteolytic activity was measured in *C. auris* cell-free extracts using specific substrates for different types of proteases. The assessed activities included acidic and neutral endopeptidases, carboxypeptidase, aminopeptidase, and dipeptidyl aminopeptidase. Extracts were obtained from yeast cultured grown under different nutritional conditions in YNB broth. YNB supplemented with dextrose and ammonium served as the control condition. Nutritional stress conditions included YNB lacking either a carbon source (YNB−C+N), a nitrogen source (YNB+C−N), or both (YNB−C−N). Also, rapamycin was used to induce autophagy.

**Figure 3:**
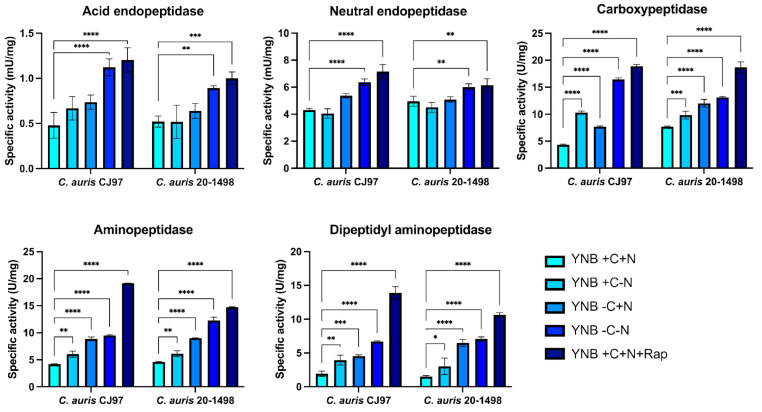
Specific proteolytic activities of cell-free extracts of *C. auris* CJ97 and 20-1498. Yeasts were cultured in different YNB media at 37 °C for six hours. The enzymatic activity of acid endoprotease, neutral endoprotease, carboxypeptidase, aminopeptidase, and dipeptidyl peptidase was assessed using denatured acid hemoglobin, hide powder azure, N-benzoyl-tyrosine-p-nitroanilide, lys-p-nitroanilide, and ala-pro-p-nitroanilide as substrates, respectively. Each bar represents the average of three independent experiments performed in triplicate, with error bars indicating the standard deviation (SD). Statistical significance was determined using two-way ANOVA: * *p* < 0.05, ** *p* < 0.01, *** *p* < 0.001, and **** *p* < 0.0001. +C: with a carbon source (2% dextrose), +N: with nitrogen source (2% NH_4_SO_4_), +Rap: with 5 nM of rapamycin, −C: without carbon source, −N: without nitrogen source.

All measured activities exhibited a significant increase in the media depleted of carbon source, with maximum activity observed in rapamycin-treated cultures compared to the control (Figure 3).

To determine the proteases responsible for each activity, enzymatic extracts were treated with specific inhibitors at different concentrations. Untreated extracts were considered to have 100% enzyme activity.

In both *C. auris* strains, 100% inhibition of acidic endopeptidases was achieved with pepstatin A at a concentration of 25 μM (Table 2). More than 70% of the neutral endopeptidase activity was inhibited by phenylmethylsulfonyl fluoride (PMSF), suggesting that the majority of the measured activity corresponds to serine peptidases. Carboxypeptidase activity was most strongly inhibited by PMSF and was least affected by E-64 and chelators, indicating that most of the activity corresponds to serine peptidases such as CpY and Atg42, followed by cysteine peptidases and metallo-carboxypeptidases such as CpS. Aminopeptidase activity was inhibited by bestatin and chelators, suggesting the presence of metallo-aminopeptidases, including Ape1 and Ape3, in the cell-free extracts. Finally, dipeptidyl aminopeptidase activity was partially inhibited by PMSF, EDTA, and bestatin, indicating that at least a portion of the detected activity corresponds to metal ion-dependent serine aminopeptidases.

### 3.3. Genes Encoding Putative Vacuolar Peptidases of C. auris Are Overexpressed Under Nutritional Stress Conditions

Out of the eight genes encoding putative vacuolar proteases, only five were selected to assess their differential expression under various nutritional stress conditions. At least one representative gene was chosen for each of the enzymatic activities measured: *PEP4*, encoding the acidic protease PrA; *PRB1*, encoding the neutral serine protease PrB; *PRC1*, encoding the carboxypeptidase CpY; *LAP4*, encoding the aminopeptidase Ape1; and *DAP2*, encoding the dipeptidyl aminopeptidase Dap2.

**Figure 4:**
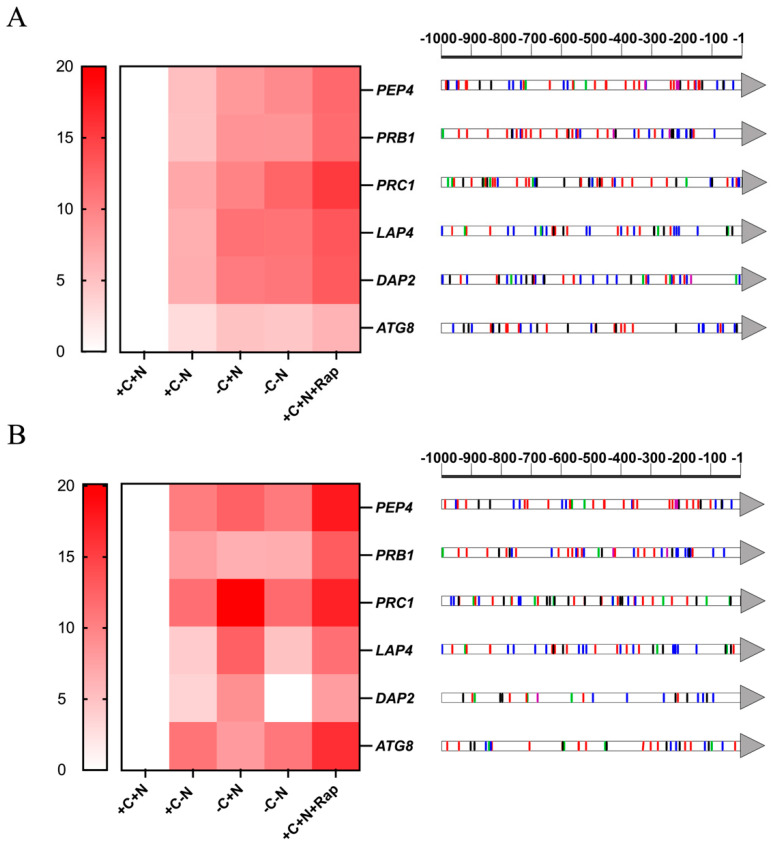
Expression levels and promoter regions of genes encoding putative vacuolar peptidases and Atg8 protein in *C. auris* CJ97 and 20-1498. In panel (**A**), strain CJ97 is illustrated, and strain 20-1498 is shown in panel (**B**). The left panel depicts the relative expression of peptidase-encoding genes and *ATG8* under different nutritional conditions. Yeast cells were incubated at 37 °C for six hours before RNA extraction in YNB media. The right panel illustrates the TFBS identified in the promoter regions of the analyzed genes. Blue: TFBS related to a carbon source, green: related to a nitrogen source, purple: related to pseudo-hyphae formation, black: related to autophagy, red: other stress responses. +C: with a carbon source (2% dextrose), +N: with nitrogen source (2% NH_4_SO_4_), +Rap: with 5 nM of rapamycin, −C: without carbon source, −N: without nitrogen source.

*C. auris* CJ97 and 20-1498 cells were grown under the same conditions used for enzyme activity measurements, and total RNA was extracted. The relative expression levels of genes encoding vacuolar peptidases, as well as the autophagy-related protein Atg8 (*ATG8*), were quantified using RT-qPCR. Gene expression in YNB+C+N medium was used as the control condition. All peptidase-encoding genes were significantly overexpressed under nutritional stress compared to the control. In general, the highest expression levels were observed under rapamycin treatment and under nutritional starvation conditions (Figure 4). In addition, *ATG8* expression was notably increased in response to nutritional depletion and rapamycin treatment.

The comparative analysis between strains revealed that *C. auris* CJ97 (clade III) exhibits lower gene expression for the studied peptidases compared to strain 20-1497 (clade IV).

On the other hand, a bioinformatic analysis was conducted to identify potential transcription factor binding sites (TFBS) within the 1000 base-pair upstream regions of the genes evaluated using RT-qPCR. The predicted TFBS in the promoter regions correlate directly with the observed gene expression levels. Thus, more TFBS are associated with increased gene expression when there is a carbon starvation, but not under nitrogen limitation. Furthermore, multiple TFBS related to starvation and autophagy response were identified, including both activators that upregulate gene expression under nutrient deprivation and repressors that downregulate expression under favorable nutritional conditions. Additionally, some TFBS linked to other stress responses, such as osmotic, thermal, and protein misfolding stress, were also predicted.

### 3.4. Vacuolar Morphology of C. auris Under Nutritional Stress and Peptidase Inhibitor Treatment

The cellular and vacuolar morphology was analyzed using transmission electron microscopy (TEM). Given that the *C. auris* 20-1498 strain (IV clade) exhibited significant overexpression of proteases-encoding genes and *ATG8* under nutrient starvation and rapamycin treatment conditions, we selected this strain for morphology visualization.

**Figure 5:**
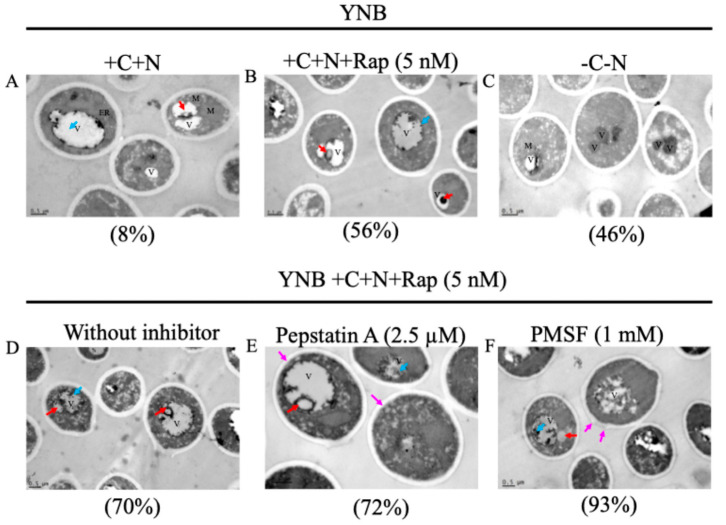
Autophagosome accumulation in *C. auris* 20-1498 (clade IV) under starvation, rapamycin treatment, and peptidase inhibitor exposure (pepstatin A and PMSF). TEM micrographs of *C. auris* 20-1498. Yeasts in panels (**A**–**C**) were incubated for 11 h at 37 °C with shaking at 100 rpm. Cells in panels (**D**–**F**) were incubated for 11 h without inhibitor under the same conditions, followed by an additional three h incubation with peptidase inhibitors. Vacuole (V), nucleus (N), mitochondrion (M), endoplasmic reticulum (ER), autophagic bodies (blue arrows), vesicles fusing to the vacuole (red arrows), and cell wall alterations (pink arrows) are indicated. The percentage of cells containing autophagic bodies is noted in parentheses below each micrograph; at least 100 cells were analyzed per condition. Scale bar = 0.5 µm. +C: with a carbon source (2% dextrose), +N: with nitrogen source (2% NH_4_SO_4_), +Rap: with 5 nM of rapamycin, −C: without carbon source, −N: without nitrogen source.

Cells cultured in YNB supplemented with carbon and nitrogen sources displayed an ovoid budding morphology with dimensions of 2.0–3.0 × 2.5–4.5 µm. The cell walls were homogeneous, measuring 200–300 nm in thickness. Vacuoles with a diameter of 1–2 µm containing electro-dense components, as well as some cytoplasmic organelles, were also observed (Figure 5A). Under nutritional starvation, cells exhibited a partially homogeneous cytoplasm, with an electro-transparent periplasm. Vacuoles were smaller, with a size of 0.33–1 µm or even undetectable, and their cytoplasm had a dense appearance. Cell size slightly decreased, although the difference was not significant (Figure 5C). Occasionally, yeast with an increased periplasmic space was also observed (Figure S6). In the treatment with rapamycin, cells with autophagic bodies were observed within the vacuole (Figure 5B). As in starvation, the cytoplasm does not appear regularly, and discrete areas of extrusion of the cytoplasmic content can be observed. The vacuole size showed a slight increase, but there was no significant difference compared to the control. Additionally, no changes were observed in cell wall thickness under any of the three conditions. On the other hand, one hundred cells were counted in different fields to estimate the number of cells containing autophagic bodies. In control, this value was 8%, whereas under starvation, it increased to 46%, and under rapamycin treatment, it reached 56%.

In *U. maydis*, inhibition of vacuolar proteases leads to the accumulation of autophagic bodies due to impaired degradation [38]. To investigate the role of peptidases in *C. auris*, we used specific inhibitors of aspartyl (pepstatin A) and serine proteases (PMSF) and analyzed vacuolar ultrastructure using TEM. *C. auris* 20-1498 cells were incubated for 11 h in YNB+C+N+Rap medium to induce autophagic bodies formation. Subsequently, cells were treated with each inhibitor separately for three h. Yeast inoculated without inhibitors served as a negative control for autophagic body accumulation.

In the control condition (without inhibitor), a partially homogeneous cytoplasm with dense bodies inside the vacuole and cytoplasmic organelles was observed. Occasionally, cytoplasmic vesicles were seen associated with the vacuole (Figure 5D). Under pepstatin A treatment, yeasts exhibited partial cytoplasmic extrusion, small membranous vesicles within the vacuole, and detachment of the cell wall (Figure 5E). PMSF treatment resulted in protrusions on the cell wall, vacuoles with distorted morphology, and a higher abundance of dense aggregates and autophagic bodies compared to other conditions (Figure 5F).

The percentages of autophagic bodies for these conditions were as follows: for the control, it was 70%, while for the condition with pepstatin A, it was 72%, and for PMSF, it was 93%. In addition, it was observed that in the condition with PMSF, there are more electrodense bodies inside the vacuole compared to the control; this was not observed with pepstatin A.

## 4. Discussion

The vacuole is a cellular compartment in fungi and plants essential for maintaining homeostasis, regulating cellular traffic, responding to different types of stress, and participating in dimorphism [39,40,41,42]. Inside the vacuolar lumen and anchored to its membrane, there are proteases with the capacity to degrade proteins and organelles [43]. In *S. cerevisiae*, vacuole proteases play a fundamental role during starvation-induced autophagy, degrading approximately 40% of total cellular proteins within the first 24 h to sustain cell survival [44].

In both pathogenic and phytopathogenic fungi, these proteases are associated with cell cycle regulation, virulence, pathogenesis, and defense against the host immune response [45,46]. In *Ustilago maydis*, the vacuolar protease PrA is required for successful infection development. Mutants deficient in this protease exhibit defects in the dimorphic transition of the mycelium and a significant reduction in virulence compared to the wild-type strain [38]. In some strains of *Trichoderma,* when the vacuolar protease B (Prb1) is secreted, it plays an important role in the mycoparasitism process, a mechanism employed as biocontrol [47]. In *Aspergillus fumigatus*, the proteins PEP2 (homolog of PrA) and ALP2 (homolog of PrB) are associated with the cell wall. Interestingly, deletion of these genes leads to a major reduction in conidial formation and growth defects. Additionally, ALP2 has been described as one of the major allergens of *A. fumigatus* [48].

Given the important role of vacuolar proteases in fungal pathogenesis, we studied the proteolytic intercellular activities associated with vacuolar proteases in *C. auris*, identifying putative encoding genes, analyzing their promoter regions, and assessing their expression under autophagy-inducing conditions.

*C. auris* is a clinically important pathogen due to its high transmissibility, global distribution, multidrug resistance, and difficulty in eradication. Since its first identification in 2009 [49], *C. auris* has been extensively studied. Genomic analyses of isolates of the different clades worldwide have revealed genetic variations that may influence gene function and strain behavior [9].

In this work, we identified eight putative orthologs of vacuolar peptidases in *C. auris* across different clades: PrA, PrB, CpY, Atg42, CpS, Ape1, Ape3, and Dap2. Phylogenetic analysis positioned *C. auris* within the CTG clade of the *Metschnikowiaceae* family, with the *Candida haemulonii* complex as its close neighbors [50]. The phylogenetics of the amino acid sequences of the eight putative vacuolar proteases in *C. auris* and related species revealed a topology consistent with previously reported evolutionary relationships for this yeast.

On the other hand, identity and similarity analyses between vacuolar peptidases from *C. auris* and *S. cerevisiae* revealed values exceeding 25% and 40%, which are established thresholds for considering proteins as homologous [51,52]. The putative vacuolar proteases of *C. auris* possess canonical domains such as signal peptides, pro-peptides, and catalytic sites. In *C. auris*, PrA, PrB, CpY, Atg42, and Ape3 contain both a signal peptide and a pro-peptide. The signal peptide directs them to the endoplasmic reticulum, where it is cleaved. The resulting pro-proteases then migrate to the Golgi apparatus and finally to the vacuole, where they mature. The pro-peptide keeps the enzyme inactive during trafficking; PrA and PrB can self-process and activate other proteases [17]. Ape1, which in *C. auris* contains only a pro-peptide, has been shown in other fungi to reach the vacuole via the cytoplasm-to-vacuole targeting (CvT) pathway, using selective autophagy [18]. CpS and Dap2 contain cytoplasmic and transmembrane domains that mediate their transport via clathrin-associated vesicles. CpS loses these domains in the vacuole, whereas Dap2 retains them, making it the only vacuolar protease anchored to the membrane [17].

In silico analysis of catalytic domains in *C. auris* putative proteases reveals structural features consistent with their classification into known peptidase families. PrA displays conserved aspartic residues and adjacent TGS motifs characteristic of A1 aspartic peptidases, which are bilobed endopeptidases active at acidic pH [53,54]. PrB exhibits the catalytic triad typical of subtilisin-like S8 serine peptidases (DHS) [55], while CpY and Atg42 show the SDH triad, consistent with serine peptidases of the S10 family [11,56]. CpS contains catalytic and zinc-binding residues indicative of the M20 metallopeptidase family, whose members coordinate two Zn^2+^ ions via conserved H/D, D, E, D/E, and H residues, and preferentially cleave amino acids adjacent to glycine [57]. Ape1 and Ape3 are also predicted metallopeptidases, with Ape1 displaying features of M18 peptidases, characterized by four catalytic and five zinc-binding residues, including E^280^ and D^331^ at the active site, which are involved in the sequential release of neutral or hydrophobic amino acids from the N-terminus [58]. Ape3 likely belongs to the M28 family, with five metal-binding residues and conserved residues E^336^ and Y^458^ involved in catalysis and transition-state stabilization [58]. Finally, Dap2 possesses the catalytic triad SDH in the typical arrangement of the S9 serine peptidase family, where serine, aspartate, and histidine act as nucleophile, electrophile, and base, respectively [11].

Protein–protein interactions were performed using the STRING tool v12.0 identifying probable proteins related to *C. auris* vacuolar proteases (Figure 2A). In this context, the putative PrA was found to be potentially associated with Kap95, a protein involved in nuclear import [59]. In *S. cerevisiae*, PrA processes the Spt-Ada-Gcn5 acetyltransferase (SAGA) complex in the nucleus, converting it into the SAGA-Like (SLIK) complex, which enhances resistance to rapamycin [60]. The predicted PrB in *C. auris* is associated with Apl1 and Apl3, proteins involved in endosomal transport, as well as with other vacuolar peptidases such as CpY. In *S. cerevisiae*, it is matured by PrB in the vacuole [17] and is inhibited by the endogenous inhibitor Tfs1, whose homolog in *C. auris* is also associated with the predicted CpY. In addition, CpY is linked to proteins involved in macroautophagy and ribophagy, such as Cdc48 [61,62]. The putative CpS is associated with Vps27, a protein involved in ubiquitination and vacuolar sorting via ESCRT (endosomal sorting complex required for transport) machinery. In *S. cerevisiae*, CpS undergoes ubiquitination as a sorting signal for its subsequent transport to endosomes destined for the vacuole [17]. Aminopeptidase Ape1 was found to interact with proteins homologous to those involved in the cytoplasm-to-vacuole targeting (CvT) pathway, such as Atg19, Ams1, and Atg11 in *S. cerevisiae* [63]. This suggests that *C. auris* may possess a CvT pathway, through which Ape1 could be transported to the vacuole. The predicted Dap2 is linked to the putative Kex2, an endoplasmic reticulum carboxypeptidase, and Pho8, a vacuolar RNase [64].

It is important to note that the enzymatic activity measurements were performed using cell-free extracts rather than vacuolar extracts. However, previous cell fractionation studies in yeasts (*S. cerevisiae* and *C. glabrata*) have shown that enzymatic activities detected in cell-free extract correlate with the vacuolar proteolytic activities [22,65]. Under nutrient-limiting conditions and rapamycin treatment, an increase in the specific activity of all tested proteases was observed: acid peptidase (aspartyl peptidase), neutral peptidase (serine peptidase), carboxypeptidase (serine peptidase), aminopeptidase (metallo-peptidase), and dipeptidyl aminopeptidase (metal-ion dependent and serine aminopeptidase), compared to the activities measured in a nutritionally complete medium (Figure 3).

Throughout this work, a comparative analysis was conducted between two *C. auris* strains from two different clades (clades III and IV, from Spain and Mexico, respectively). The clade III strain exhibited higher intracellular enzymatic activity than the clade IV strain. Additionally, the expression levels of genes encoding vacuolar proteases were higher in the Mexican strain compared to the Spanish strain. This finding is consistent with the well-documented clade-specific characteristics of *C. auris*, which include differences in metabolism, antifungal resistance, accessory proteins, virulence, and stress response profiles [1]. Isolates from different clades of *Candida auris* differ by tens of thousands of single-nucleotide polymorphisms (SNPs), whereas the number of SNPs within each clade is minimal, suggesting a series of clonal expansions [66]. SNPs also impact critical functions such as enzymatic activity and gene regulation. For example, mutations in the *ERG11* gene, such as Y132F and K143R, alter the structure of lanosterol 14-α-demethylase, reducing its affinity for azoles and contributing to antifungal resistance [67]. Similarly, SNPs in transcription factors like TAC1B can lead to the overexpression of efflux pumps, such as CDR1, increasing drug efflux and promoting multidrug resistance [66]. These genetic modifications, found across different clades and strains, reflect the adaptive capacity of *C. auris*.

The differential expression of the genes *PEP4*, *PRB1*, *PRC1*, *LAP4*, and *DAP2* was assessed because they are the most extensively studied vacuolar protease genes and have been linked to autophagy. Additionally, their protein products are considered canonical vacuolar hydrolases [18]. These genes were also selected based on previous findings from our research group, which demonstrated their involvement in nutritional stress responses in other yeasts of medical and phytosanitary relevance [14,22,38].

Under nutrient-limiting conditions and rapamycin treatment, we observed increased expression of transcripts encoding the peptidases PrA (*PEP4*), PrB (*PRB1*), CpY (*PRC1*), Ape1 (*LAP4*), and Dap2 (*DAP*2) compared to the control (Figure 4). Additionally, under these conditions, we also observed overexpression of the gene encoding the Atg8 protein, which is noteworthy because Atg8 is commonly used as a marker of autophagy in yeast cells [68]. In *S. cerevisiae*, vacuolar peptidases play a critical role in the cellular response and survival under nutrient starvation and act as executing enzymes in the late stages of autophagy [44,69,70,71]. Similarly, in *C. albicans*, the genes *Apr1* (orthologue of *PEP4*) and *Cpy1* (orthologue of *PRC1*) are upregulated under nitrogen-limiting conditions, while their expression is nearly undetectable in complete media [72]. In *C. glabrata*, the predicted proteases genes *PEP4*, *PRB1*, *APE1*, and *APE3* showed increased expression and enzymatic activity under autophagy-inducing conditions [14,22]. Promoter regions analysis in *C. glabrata* revealed TFBS belonging to the NIT2 (activator of nitrogen-regulated genes) family but lacked CSER (carbon source response elements) elements [14]. Interestingly, in *C. auris*, a greater number of TFBS associated with the response to the carbon source were identified within 1000 bp upstream of the eight analyzed genes. This correlates with the observed gene expression pattern, where expression levels were higher under carbon-limiting conditions than under nitrogen-limiting conditions. However, TFBS analyses should be interpreted cautiously, as they were based on known *S. cerevisiae* elements, and *C. auris* may possess uncharacterized TFBS. Future transcriptomic analyses of *C. auris* under nutritional stress conditions will be essential to elucidate its survival mechanisms, particularly given its persistence in nosocomial environments, where it can colonize both patient skin and abiotic surfaces such as tables and catheters [73].

Under the aforementioned conditions of starvation and rapamycin treatment, in addition to the increased expression of *ATG8* and protease-related genes, autophagic bodies were detected, with a higher frequency observed under rapamycin treatment (Figure 5), suggesting that these conditions induce autophagy in *C. auris*. Additionally, under starvation conditions, yeast cells exhibited smaller vacuoles with dark, electron-dense content, increased periplasmic space, and a denser cytoplasm (Figure 5 and Figure S6) compared to cells in nutrient-rich conditions. In addition to the increased autophagy and protease activity, these morphological changes suggest that nutrient scarcity may induce a reprogramming in *C. auris* to promote survival. Previous studies have shown that during the stationary phase, when nutrients are exhausted, yeast cells typically enter a quiescent state characterized by morphological, enzymatic, and general gene expression response [74,75,76,77].

Since the inhibition or deletion of key vacuolar peptidases, such as PrA and PrB, affects autophagy in other fungi, we sought to investigate the role of these enzymes in *C. auris* by inhibiting PrA and PrB using pepstatin A and PMSF, respectively, before autophagy induction by rapamycin. Treatment with pepstatin A resulted in cell wall detachment and the presence of autophagic bodies, without any intracellular alterations beyond those observed in the rapamycin-treated control. Notably, pepstatin A inhibits mTORC1 in human cells and its orthologs in yeasts, thereby activating autophagy [78]. Furthermore, cell wall alterations were observed, which may be linked to the presence of yapsins, a family of wall-anchored aspartyl peptidases found in *C. glabrata* that are essential for maintaining cell wall integrity and are known targets of pepstatin A [79,80]. Moreover, GPI (glycosylphosphatidylinositol)-linked aspartyl wall proteases regulate vacuole homeostasis in *C. glabrata* [81]. This suggests a potential indirect relationship between yapsins and the vacuolar peptidases, which warrants further investigation.

Treatment with PMSF, an irreversible inhibitor of serine peptidase such as PrB, led to the accumulation of electrodense bodies in the vacuolar lumen and protrusions in the *C. auris* cell wall. A similar phenotype was observed in *C. glabrata*, where a serine peptidase anchored to the cell wall by β-1,3 glycosidic bonds exhibits gelatinolytic activity and is inhibited by PMSF, suggesting that wall-associated peptidases play a role in shaping the cell wall [82]. Moreover, PMSF has been shown to induce the accumulation of autophagic bodies in *S. cerevisiae* and *U. maydis*, preventing their degradation over time due to the inhibition of proteases responsible for their clearance [38,71]

**Figure 6:**
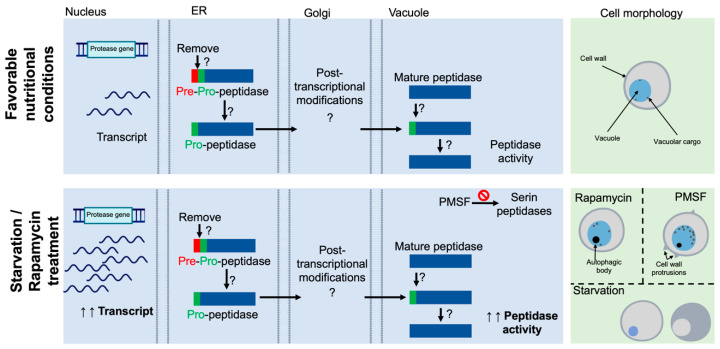
Model of *C. auris* vacuolar peptidases and autophagy. *C. auris* encodes putative vacuolar proteases, including the aspartyl endopeptidase PrA (*PEP4*), the serine endopeptidase PrB (*PRB1*), the serine carboxypeptidase CpY (*PRC1*), the metalloaminopeptidase Ape1 (*LAP4*), and the dipeptidyl aminopeptidase Dap2 (*DAP2*). The enzymes exhibit domains characteristic of vacuolar proteases described in *S. cerevisiae*. These hydrolases are synthesized as pre-pro-peptidases, where the “pre” segment refers to a signal peptide that targets the protein to the endoplasmic reticulum (ER). Once in the ER, the signal peptide is cleaved, producing the pro-peptidase form, which may then transit through the Golgi apparatus, presumably undergoing post-translational modifications before being trafficked to the vacuole. Within the vacuole, mature proteases cleave the pro-peptide, activating the enzyme. The mature forms exhibit proteolytic activity required for protein degradation in the vacuolar lumen. The expression of these genes, as well as the proteolytic activity, is basal under nutrient-rich conditions but increases in response to nutritional stress and rapamycin treatment. These conditions also induce morphological changes: under starvation, small vacuoles and the cytoplasm become contracted and electron-dense, while under rapamycin treatment, cells accumulate autophagic bodies. Furthermore, inhibition of vacuolar serine proteases with PMSF also causes accumulation of autophagic bodies and alterations in cell wall architecture, including the appearance of protrusions. Black arrows with a question mark indicate theoretical bioinformatic predictions.

In conclusion, our findings indicate that *C. auris* possesses genes encoding putative vacuolar proteases orthologs to those in *S. cerevisiae* and other fungi. The analysis of their predicted domains and motifs, interactome, tertiary structure, and ligands suggests that these enzymes likely participate in vacuolar trafficking and maturation pathways. Under starvation and rapamycin treatment, we observed increased intracellular proteolytic activity and the overexpression of genes encoding vacuolar peptidases, along with *ATG8*, a well-established autophagy marker, compared to control conditions. Furthermore, the accumulation of autophagic bodies within vacuoles under nutritional starvation, rapamycin treatment, and protease inhibition, along with alterations in cell wall morphology, supports the involvement of *C. auris* vacuolar proteases in the autophagy process (Figure 6).

## Figures and Tables

**Table 1:** Characteristics of the genes and their predicted vacuolar proteases of *C. auris*.

Gen/Protein	*C. auris* CJ97 (Clade III)		*C. auris* 20-1498 (Clade IV)	
	Gene Length (bp)	Protein Length (aa)/Molecular Mass (kDa)	Gene Length (bp)	Protein Length (aa)/Molecular Mass (kDa)
*PEP4*/PrA	1230	409/44.33	1230	409/44.33
*PRB1*/PrB	1632	543/57.4	1632	543/57.4
*PRC1*/CpY	1629	542/61.42	1629	542/61.42
*ATG42*/Atg42	1707	568/63.74	1707	568/63.76
*CPS*/CpS	1635	544/61.28	1635	544/61.29
*LAP4*/Ape1	1383	460/50.61	1383	460/50.5
*APE3*/Ape3	1572	523/57.37	1572	523/57.379
*DAP2*/Dap2	2379	842/95.68	2379	792/89.98

**Table 2:** Specific inhibition profile of intracellular proteolytic activities in *C. auris*.

		Residual Activity (%)	
Enzymatic Activity (Catalytic Type)	Inhibitors (Concentration)	*C. auris* 20-1498	*C. auris* CJ97
**Acidic proteinase** (aspartyl peptidase)	Pepstatin A: 2.5/5/25 μM	36.66/25.11/0.0	59.53/0.0/0.0
**Neutral proteinase** (serine peptidase)	PMSF: 1/5 mM	45.6/33.23	66.56/37.8
**Carboxypeptidase** (serine peptidase)	PMSF: 1/5 mM	46.44/36.82	32.71/21.7
	EDTA: 1/10 mM	95.39/78.67	76.23/69.44
	E-64: 1/10 μM	96.4/41.99	100/35.63
**Aminopeptidase** (metallo-aminopeptidase)	Bestatin: 100/250 μM	94.47/63.87	93.71/59.01
	EDTA: 1/10 mM	100/72. 05	90.75/57.63
	1,10 phenanthroline: 2.5/7.5 mM	80.49/23.25	81.66/19.16
**Dipeptidyl aminopeptidase** (metal-ion-dependent and serine aminopeptidase)	PMSF: 1/5 mM	100/67.17	100/83.67
	EDTA: 1/10 mM	59.7/55.73	40.06/32.5
	E-64: 1/10 mM	100/100	100/100
	Bestatin: 100/250 μM	100/32.83	100/28.11

## Data Availability

The original contributions presented in this study are included in the article/Supplementary Materials. Further inquiries can be directed to the corresponding authors.

## Supplementary Materials

The following supporting information can be downloaded at: https://www.mdpi.com/article/10.3390/jof11050388/s1, Figure S1: Analysis of PrA and PrB sequences. Figure S2: Analysis of CpY andAtg42 sequences. Figure S3: Analysis of CpS and Ape1 sequences. Figure S4: Analysis of Ape3 and Dap2 sequences. Figure S5: Superposition of the tertiary structures of the proteases PrA, CpY and Ape1 from *S. cerevisiae* (yellow) and *C. auris* (blue). Figure S6: TEM micrograph of *C. auris* 20-1498 (clade IV) under starvation of carbon and nitrogen sources, showing increased periplasmic space (Red arrows).
