# Supplementary material for: Vacuolar Proteases of Candida auris from Clades III and IV and Their Relationship with Autophagy
Source: J Fungi (Basel). 2025 May 18;11(5):388. doi: 10.3390/jof11050388 (PMC12113386; doi:10.3390/jof11050388)
Supplement: Supplementary file 1 [file jof-11-00388-s001.zip › jof-3592871-supplementary.pdf]

Supplementary material

Vacuolar proteases of *Candida auris* from clades III and IV and their relationship with autophagy

A

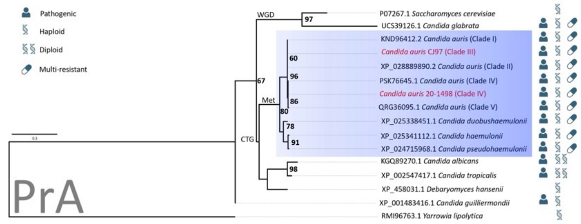

B

C. auris	1	2	3	4	5	6
1. 6686		99.8	99.5	99	100	99.5
2. B11220	100		99.3	98.8	99.8	99.3
3. B11243	100	100		99.5	99.5	100
4. IFRC2087	100	100	100		99	99.5
5. CJ97	100	100	100	100		99.5
6. 20-1498	100	100	100	100	100	

C

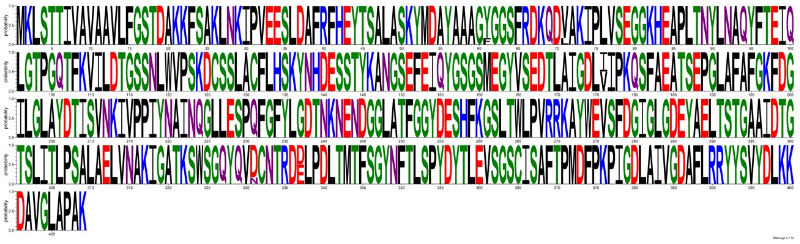

A

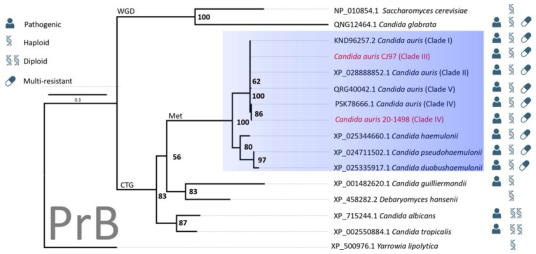

B

C. auris	1	2	3	4	5	6
1. 6686		99.8	99.4	99.3	99.8	99.4
2. B11220	100		99.6	99.4	100	99.6
3. B11243	100	100		99.1	99.6	100
4. IFRC2087	99.8	99.8	99.8		99.4	99.1
5. CJ97	100	100	100	99.8		99.6
6. 20-1498	100	100	100	99.8	100	

C

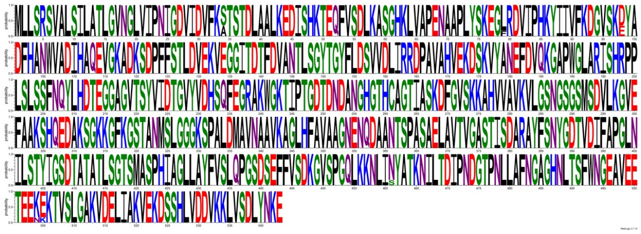

Figure S1. Analysis of PrA and PrB sequences. (**A**) PrA phylogeny of *C. auris* from different clades and related species of the WGD and CTG clade. The substitution model employed was WAG+G4 (-4831,210) with 1000 bootstrap was used for phylogenetic reconstruction. The family Metschnikowiaceae is highlighted in blue. Only Bootstrap values >50 is shown. (**B**) percentages of similarity (red) and identity (blue) between the different strains of *C. auris*. (**C**) Logo between the sequences of the different *C. auris* strains. Neutral (purple), acidic (red), basic (blue), polar (green) and hydrophobic (black) amino acids are illustrated in the analysis. (**D**) PrB phylogeny of *C. auris* from different clades and related species of the WGD and CTG clade. The substitution model employed was LG+F+I+G4 (-6401.887) with 1000 bootstrap was used for phylogenetic reconstruction. (**E**) Percentages of similarity and identity between the different strains of *C. auris*. (**F**) Logo between the sequences of the different *C. auris* strains. Met: Metschnikowiaceae. WGD: whole genome duplication.

A

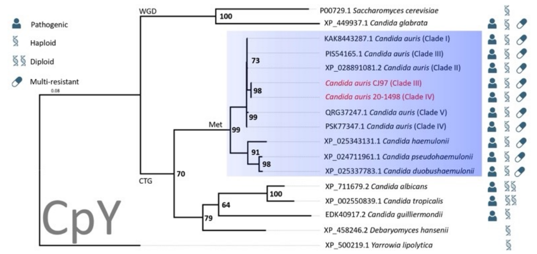

B

C. auris	1	2	3	4	5	6
1. B8441		95.8	95.8	99.4	98.9	98.5
2. B11220	95.8		99.6	95.4	94.8	94.6
3. B11243	95.9	99.8		95.6	94.8	95
4. IFRC2087	99.6	95.4	95.6		98.3	98.2
5. CJ97	98.9	94.8	95	98.5		99.6
6. 20-1498	98.7	94.8	95	98.3	99.8	

C

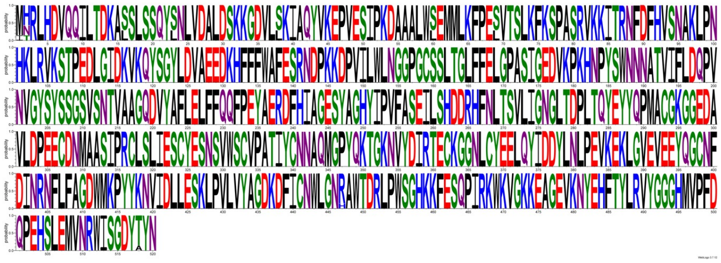

A

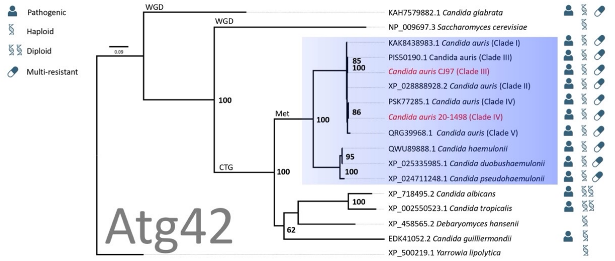

B

C. auris	1	2	3	4	5	6
1. B8441		99.6	99.3	98.3	99.4	99.1
2. B11220	99.6		99.3	98.3	99.4	99.1
3. B11243	99.6	99.6		98.3	99.1	99.8
4. IFRC2087	99.1	99.1	99.4		98.2	98.2
5. CJ97	99.4	99.4	99.4	98.9		99.3
6. 20-1498	99.4	99.4	99.8	99.3	99.6	

C

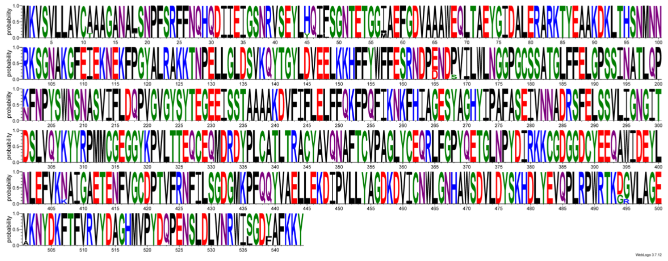

Figure S2. Analysis of CpY and Atg42 sequences. (**A**) CpY phylogeny of *C. auris* from different clades and related species of the WGD and CTG clade. The substitution model employed was WAG+I+I+R2 (-7421.179) with 1000 bootstrap was used for phylogenetic reconstruction. The family Metschnikowiaceae is highlighted in blue. Only Bootstrap values >50 is shown. (**B**) percentages of similarity (red) and identity (blue) between the different strains of *C. auris*. (**C**) Logo between the sequences of the different *C. auris* strains. Neutral (purple), acidic (red), basic (blue), polar (green) and hydrophobic (black) amino acids are illustrated in the analysis. (**D**) Atg42 phylogeny of *C. auris* from different clades and related species of the WGD and CTG clade. The substitution model employed was WAG+F+I+G4 (-7299.928) with 1000 bootstrap was used for phylogenetic reconstruction. (**E**) Percentages of similarity and identity between the different strains of *C. auris*. (**F**) Logo between the sequences of the different *C. auris* strains. Met: Metschnikowiaceae. WGD: whole genome duplication.

A

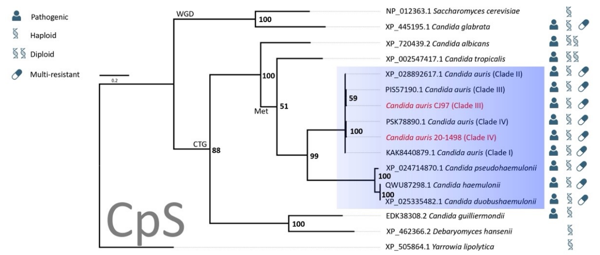

B

C. auris	1	2	3	4	5	6
1. B8441		99.6	99.1	45.8	98.4	98.4
2. B11220	100		99.5	46	98.8	98.8
3. B11243	100	100		45.5	98.2	99.3
4. IFRC2087	66.1	66.1	66.1		46.3	45.8
5. CJ97	99.1	99.1	99.1	66.4		98.9
6. 20-1498	99.3	99.3	99.3	66.6	99.8	

C

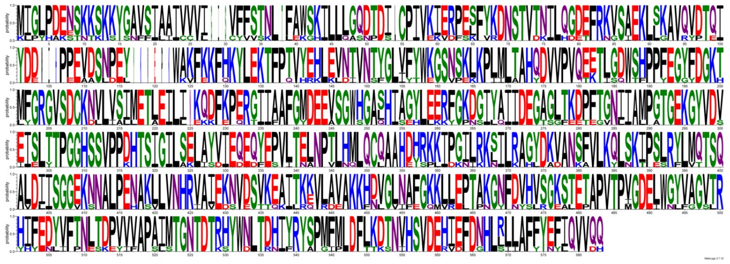

A

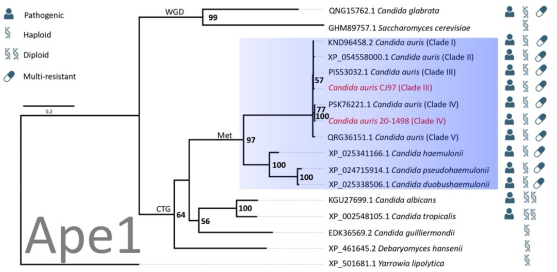

B

C. auris	1	2	3	4	5	6
1. 6686		96.3	98.9	98.7	99.3	98.5
2. B11220	96.5		95.7	95.7	96.1	95.2
3. B11243	99.8	96.3		98.5	98.7	99.6
4. IFRC2087	99.6	96.3	99.3		98.5	98
5. CJ97	99.6	96.1	99.3	99.1		99.1
6. 20-1498	99.3	95.9	99.6	98.9	99.8	

C

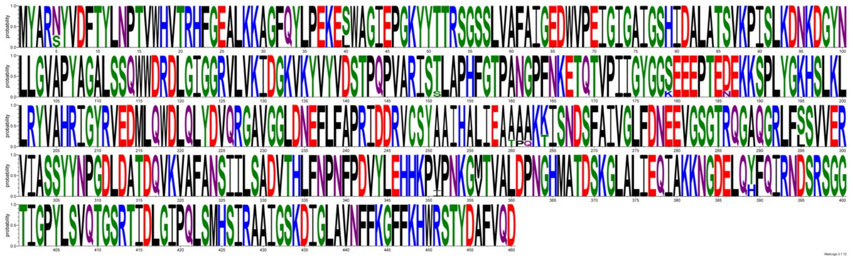

Figure S3. Analysis of CpS and Ape1 sequences. (**A**) CpS phylogeny of *C. auris* from different clades and related species of the WGD and CTG clade. The substitution model employed was LG+F+I+G4 (-9623.317) with 1000 bootstrap was used for phylogenetic reconstruction. The family Metschnikowiaceae is highlighted in blue. Only Bootstrap values >50 is shown. (**B**) Percentages of similarity (red) and identity (blue) between the different strains of *C. auris*. (**C**) Logo between the sequences of the different *C. auris* strains. Neutral (purple), acidic (red), basic (blue), polar (green) and hydrophobic (black) amino acids are illustrated in the analysis. (**D**) Ape1 phylogeny of *C. auris* from different clades and related species of the WGD and CTG clade. The substitution model employed was Q.yeast+I+I+R2 (-6045.352) with 1000 bootstrap was used for phylogenetic reconstruction. (**E**) Percentages of similarity and identity between the different strains of *C. auris*. (**F**) Logo between the sequences of the different *C. auris* strains. Met: Metschnikowiaceae. WGD: whole genome duplication.

A

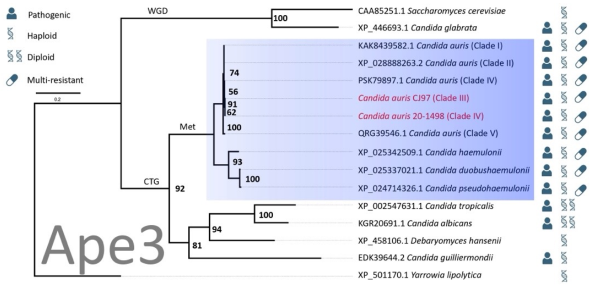

B

C. auris	1	2	3	4	5	6
1. 6686		99.8	99.8	98.9	99.8	99.6
2. B11220	100		99.6	98.7	99.6	99.4
3. B11243	100	100		98.7	99.6	99.8
4. IFRC2087	99.6	99.6	99.6		98.7	98.5
5. CJ97	99.8	99.8	99.8	99.4		99.8
6. 20-1498	99.8	99.8	99.8	99.4	100	

C

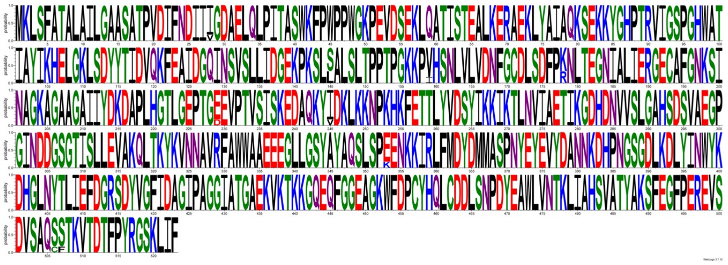

A

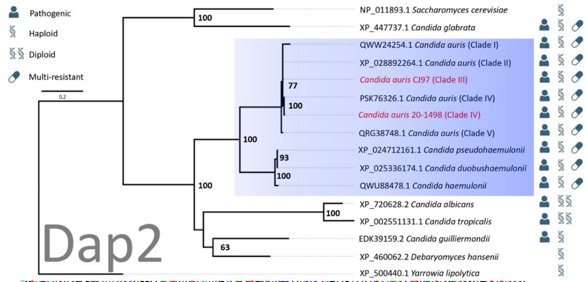

B

C. auris	1	2	3	4	5	6
1. CA7LBN		85	84.2	83.8	84.8	84
2. B11220	86.1		99.1	98.6	99.7	98.9
3. B11243	85.8	99.6		98	98.9	99.7
4. IFRC2087	85.8	99.6	99.2		98.4	97.7
5. CJ97	85.9	99.7	99.4	99.4		99.1
6. 20-1498	85.6	99.4	99.7	99	99.6	

C

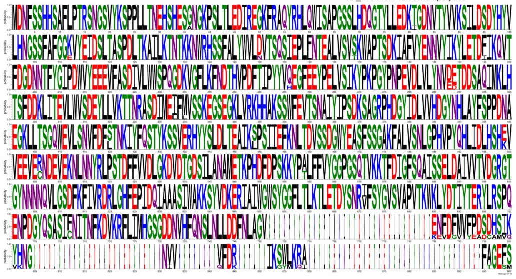

Figure S4. Analysis of Ape3 and Dap2 sequences. (**A**) Ape3 phylogeny of *C. auris* from different clades and related species of the WGD and CTG clade. The substitution model employed was LG+F+I+G4 (-7077.873) with 1000 bootstrap was used for phylogenetic reconstruction. The family Metschnikowiaceae is highlighted in blue. Only Bootstrap values >50 is shown. (**B**) Percentages of similarity (red) and identity (blue) between the different strains of *C. auris*. (**C**) Logo between the sequences of the different *C. auris* strains. Neutral (purple), acidic (red), basic (blue), polar (green) and hydrophobic (black) amino acids are illustrated in the analysis. (**D**) Dap2 phylogeny of *C. auris* from different clades and related species of the WGD and CTG clade. The substitution model employed was cpREV+F+I+G4 (-12022.322) with 1000 bootstrap was used for phylogenetic reconstruction. (**E**) Percentages of similarity and identity between the different strains of *C. auris*. (**F**) Logo between the sequences of the different *C. auris* strains. Met: Metschnikowiaceae. WGD: whole genome duplication.

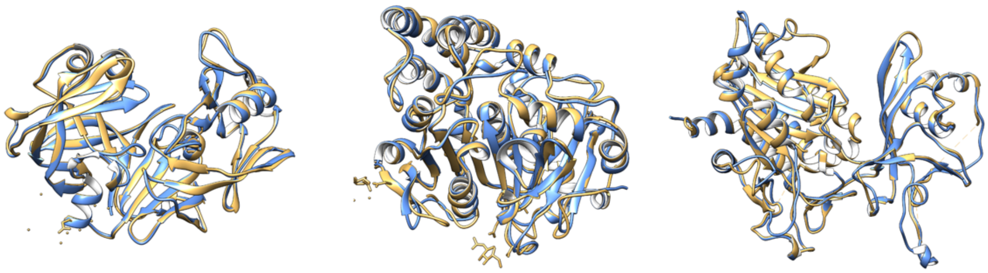

Figure S5. Superposition of the tertiary structures of the proteases PrA, CpY and Ape1 from *S. cerevisiae* (yellow) and *C. auris* (blue).

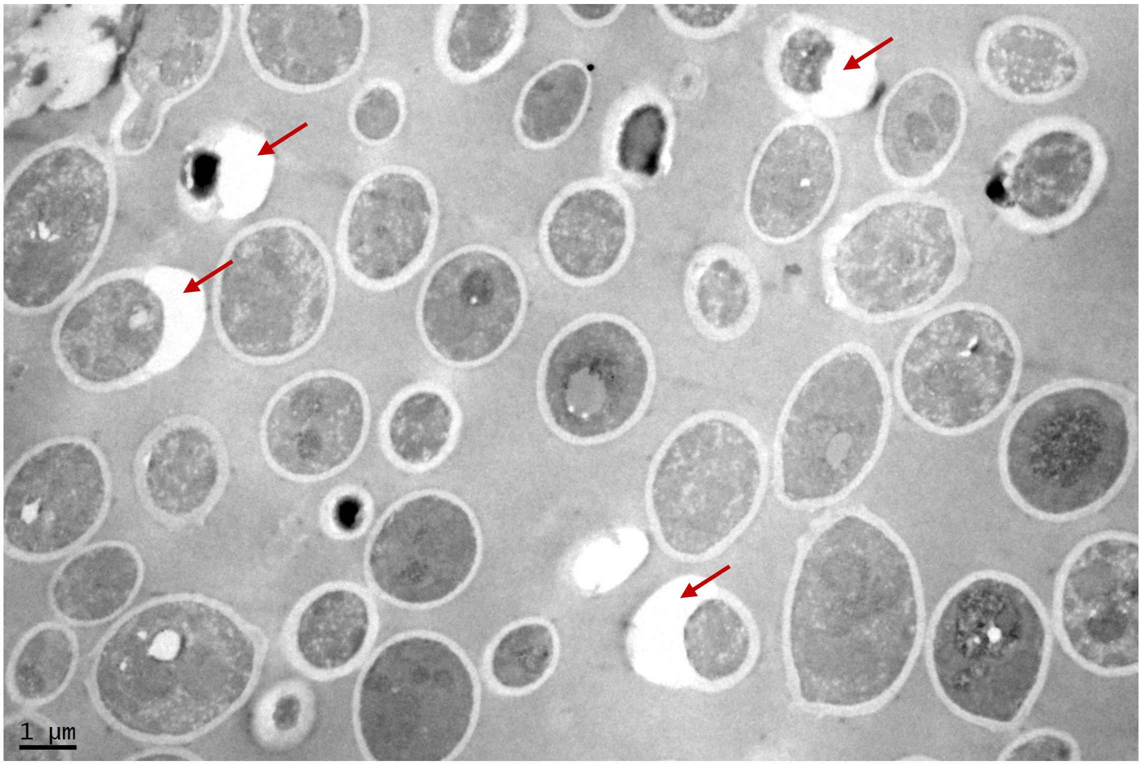

Figure S6. TEM micrograph of *C. auris* 20-1498 (clade IV) under starvation of carbon and nitrogen sources, showing increased periplasmic space (Red arrows).
